# Gender-based violence in conflict and displacement: qualitative findings from displaced women in Colombia

**DOI:** 10.1186/1752-1505-8-10

**Published:** 2014-07-11

**Authors:** Andrea L Wirtz, Kiemanh Pham, Nancy Glass, Saskia Loochkartt, Teemar Kidane, Decssy Cuspoca, Leonard S Rubenstein, Sonal Singh, Alexander Vu

**Affiliations:** 1 Department of Emergency Medicine, Johns Hopkins Medical Institute, Baltimore, USA; 2 Center for Public Health and Human Rights, Department of Epidemiology, Johns School of Public Health, Baltimore, USA; 3 Department of International Health, Johns Hopkins School of Public Health, Baltimore, USA; 4 Johns Hopkins School of Nursing, Baltimore, USA; 5 United Nations High Commissioner for Refugees, Bogota, Colombia; 6 Departamento de Antropología de la Universidad de los Andes, Bogota, Colombia; 7 Department of General Internal Medicine, Johns Hopkins Medical Institute, Baltimore, USA

**Keywords:** Gender-based violence, Intimate partner violence, Conflict, Displacement, Colombia, Humanitarian settings

## Abstract

**Introduction:**

Gender-based violence (GBV) is prevalent among, though not specific to, conflict affected populations and related to multifarious levels of vulnerability of conflict and displacement. Colombia has been marked with decades of conflict, with an estimated 5.2 million internally displaced persons (IDPs) and ongoing violence. We conducted qualitative research to understand the contexts of conflict, displacement and dynamics with GBV. This as part of a multi-phase, mixed method study, in collaboration with UNHCR, to develop a screening tool to confidentially identify cases of GBV for referral among IDP women who were survivors of GBV.

**Methods:**

Qualitative research was used to identify the range of GBV, perpetrators, contexts in conflict and displacement, barriers to reporting and service uptake, as well as to understand experiences of service providers. Thirty-five female IDPs, aged 18 years and older, who self-identified as survivors of GBV were enrolled for in-depth interviews in San Jose de Guaviare and Quibdo, Colombia in June 2012. Thirty-one service providers participated in six focus group discussions and four interviews across these sites.

**Results:**

Survivors described a range of GBV across conflict and displacement settings. Armed actors in conflict settings perpetrated threats of violence and harm to family members, child recruitment, and, to a lesser degree, rape and forced abortion. Opportunistic violence, including abduction, rape, and few accounts of trafficking were more commonly reported to occur in the displacement setting, often perpetrated by unknown individuals. Intrafamilial violence, intimate partner violence, including physical and sexual violence and reproductive control were salient across settings and may be exacerbated by conflict and displacement. Barriers to reporting and services seeking were reported by survivors and providers alike.

**Conclusions:**

Findings highlight the need for early identification of GBV cases, with emphasis on confidential approaches and active engagement of survivors in available, quality services. Such efforts may facilitate achievement of the goals of new Colombian laws, which seek to prevent and respond to GBV, including in conflict settings. Ongoing conflict and generalized GBV in displacement, as well as among the wider population, suggests a need to create sustainable solutions that are accessible to both IDPs and general populations.

## Introduction

Gender-based violence (GBV) is a global public health problem and a violation of human rights. Broadly defined by the United Nations, GBV is any violent act that is perpetrated on the basis of socially-ascribed gender differences. This umbrella term encompasses those types of intimate partner violence (IPV) and non-partner rape, as well as a range of violent acts including other physical, psychological, economic, and sexual violence, exploitive or coercive acts, as well as a harmful traditional practices [1]. GBV experiences may occur within intimate relationships, families, and may also extend to perpetration by others beyond the household, including known or unknown individuals [2]. Most recently, global estimates suggest that 35% of women around the world experience some form of sexual or physical intimate partner or non-partner violence over the course of their lives [3]. Likewise, a multi-country cross-sectional study of men in Asia and the Pacific has estimated that 25 to 87% of men have committed lifetime perpetration of IPV while 2 to 26% have perpetrated non-partner rape across these settings [4,5].

In the last decade, the problem of GBV in conflicts and its devastating consequences on the lives of conflict-affected persons has been documented extensively [2,6-11]. GBV has been acknowledged as a weapon of war or conflict, often used as a means to control and intimidate a population, but is a highly heterogeneous phenomenon in its prevalence and perpetrators [9]. Most research has documented violations of women and girls, demonstrating rape in war, and other types of violence, including child sexual abuse, forced or coerced prostitution or trafficking, and other forms of sexual exploitation [12]. IPV has also been documented among conflict-affected populations, including refugees and internally displaced persons (IDPs), and stress related to conflict may be a trigger for IPV or may exacerbate ongoing violence [13-15]. Similar forms of violence experienced by men and boys in conflict affected populations have also begun to be documented [16,17]. Refugees and IDPs are particularly vulnerable to GBV: conflict, forced displacement of populations, separation of families, disruption of community and institutional protection structures, create opportunities for GBV perpetration and challenge access to justice for survivors [2]. Perpetration of GBV may also occur during transit of conflict-affected populations and/or refuge and may be perpetrated by a range of actors [7,18,19]. GBV can lead to severe physical, reproductive, and mental health sequelae [3,20-22]. While these health outcomes also occur in non-conflict settings, they may be exacerbated in conflict settings by a lack of access to or improper medical care, concurrent infectious disease, malnutrition, stress, and other health problems. [2]

These experiences of conflict, displacement, and GBV have been documented in the last two decades of the ongoing conflict in Colombia [23-25]. Political conflict and violence began in the 1940’s and from this the current conflict between the Colombian Government and other armed groups emerged in the 1960’s. Since then, Colombia has faced a protracted conflict between the Colombian Government and a variety of illegal armed groups, including the two guerrilla groups that remain active in the country- the Fuerzas Armadas Revolucionarias de Colombia (FARC), and the Ejército de Liberación Nacional (ELN). Conflict, territorial control, and gross human rights violations have also been attributed to several paramilitary groups, despite demobilization of one of the largest right-wing paramilitary, as well as criminal gangs, referred to as *Bacrim*, which may include some demobilized members of the paramilitary [23,24]. These groups routinely commit human rights violations to maintain their control over territory and civilian populations [24,25]. Whether displacement of civilians is purposely forced or as a consequence of violence between the various armed groups, Colombia has become a country with the highest number of IDPs across the globe: as of December 2013, an estimated 5.2 million people are displaced within the country [26]. Most IDPs have been displaced from rural to urban areas; yet, violence in larger urban centers has led to substantial intra-urban displacement, signifying a shift in displacement modalities [27]. Urban violence has been attributed to clashes between illegal armed groups and government forces, activities of post-demobilization groups, disputes over the control of urban areas that include profitable micro-drug trade, forced recruitment or labor, and pressure on communities to engage in illegal mining and illicit plant cultivation [24,27].

Among refugees and IDP populations, survivors must actively seek treatment, healthcare, and/or justice and protection for GBV. For a number of reasons, however, including shame, stigma, low awareness of or access to services, and impunity, GBV is often under-reported and available services under-utilized [28]. The research presented here is part of a larger study that attempts to overcome under-reporting and under-utilization of services among IDP and refugee populations [18]. The qualitative research presented here was a collaboration with the United Nations High Commissioner for Refugees (UNHCR) and aimed to ultimately inform the development of a screening tool to confidentially identify GBV among female IDPs in Colombia for referral to GBV services.

## Methods

Qualitative research was conducted to identify the range of GBV experiences, perpetrators, and the contexts in which GBV occurs in conflict and displacement. Additional research sought to understand barriers to survivors’ disclosure and reporting of GBV as well as experiences of service providers who respond to such settings. Development of the qualitative research protocol and semi-structured interview guides was informed by previous research of GBV among conflict-affected populations [18], a preliminary review of GBV and sexual violence among conflict-affected populations [6], and preliminary consultation with the UNHCR, the Ministry of Social Protection, and IDPs and civil society community organizations based in Bogota, Soacha, and Mocoa.

### Setting and participants

**Figure 1:**
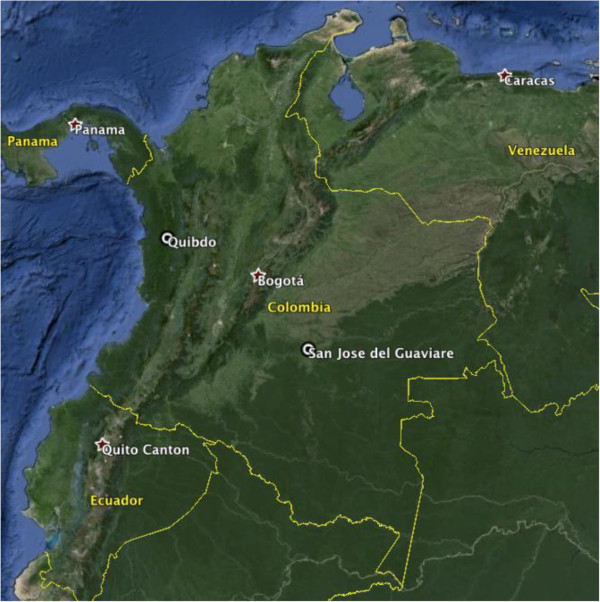
**Colombia country map and data collection sites, Quibdo and San Jose de Guaviare.** Figure created by Google Earth; sources: US Dept of State Geographer, 2013 Google, SIO, NOAA, US Navy, NGA, Landsat.

Research was conducted in San Jose de Guaviare and Quibdo, Colombia in June 2012 (Figure 1). San Jose de Guaviare is located in the department of Guaviare, which is a rural department well known for conflict and displacement. San Jose de Guaviare is a small town in which the majority of the population is now comprised of displaced persons who have relocated from more remote areas. Quibdo, in the department of Chocó, is an urban area located near the Pacific coast, receives displaced populations and also suffers intra-urban violence and displacement. All study activities were conducted in private offices of local community-based and humanitarian service providers (names excluded for security purposes) in San Jose de Guaviare and in Quibdo.

Thirty-five female IDPs who self-identified as survivors of GBV were enrolled for in-depth interviews. Inclusion criteria for female survivors included: female gender, aged 18 years or older, self-reported status as internally displaced (legal proof not required), had reported and was/currently receiving services for GBV from a collaborating organization. Women judged to be cognitively impaired or too traumatized to participate were excluded from the study. Thirty-one service providers who worked in fields providing psychosocial programs for GBV experiences, health services, protection and response to GBV, women’s programs, and humanitarian programs participated in six focus group discussions and four interviews across these two sites. Table 1 displays the number of participants enrolled per site.

**Table 1:** Qualitative study participation and demographic characteristics by site and participant type

**Survivor Interviews**	
**Site:**	N
**Guaviare**	23
**Quibdo**	12
**Total Survivor Interviews:**	30
**Mean Age, yrs. (range):**	39 (18-61 yrs)
**Provider Focus Group Discussions and Interviews**	
**Site:**	N
**Guaviare**	3 groups
**Quibdo**	3 groups
**Total Focus Group Discussions**	6 groups
**Provider Interviews (Quibdo)**	4
**Total Provider Participants**	31
**Service organizations represented**:	
*Health and Psychosocial:* Capricon; Ministry of Health (Municipal); Personería; Hospital de San José; Ministerio de Salud (Departamental); Instituto Colombiano de Bienestar Familial; Ismael Roldan Hospital; San Francisco de Assis; Hospital; Instituto Colombiano Bienestar Familial;	
*Humanitarian:* International Committee of the Red Cross; UNHCR;	
*Protection:* Defensoría (ombudsman); Fiscalía (prosecutor’s office);	
*Community:* Ruta Pacífica; Cocomancia; Red Departmental de Mujeres Chocoanes; Fundación Semilla de Vhida Positiva; Diocese	

### Recruitment and consent

Female survivors were invited to participate in research by local organizations providing GBV-related services. Likewise, service providers were invited during preliminary meetings and through outreach by UNHCR to participate in focus group discussions. To prevent unintentional disclosure of a participant’s involvement in the project, neither study fliers, nor general announcements of the project were circulated among the IDP populations to “advertise” the project.

Eligible participants of the interviews and focus group discussions were informed of all aspects of the project, including purpose, risks, benefits, and all study safety measures to protect research participants. Participants were informed that none of their personal information would be recorded and that services received from partner agencies would not be affected by participation in or refusal to participate in the study. No incentives were provided for participation. Eligible participants were asked for verbal consent prior to participation in the study. Verbal consent processes were used in lieu of written consent to prevent any linkage of a participant’s name to the study. Participants were offered copies of the consent form but were not required to take the form, unless desired by the participant. Participants were also asked for separate consent to digitally record interviews and discussions and were informed of security measures in place to protect recordings. Two survivors declined to be recorded; data for these interviews were collected by hand written notes. GBV service providers who wished to participate in the project, but who did not feel comfortable talking in groups were invited to complete an individual interview using the same semi-structured guide and protocol as was implemented in the focus group discussions. Participant confidentiality was maintained throughout the entire consent and discussion/interview processes and participants of the discussions were specifically requested to respect the confidentiality of other participants in the groups.

### Qualitative measures

In-depth interviews and focus group discussions were implemented by three experienced research team members (AW, KP, and GJ) with the use of semi-structured interview guides. Key domains of interviews and discussions included: 1) types and contexts of GBV experienced by IDPs; 2) perpetrators (e.g. husband, family member, neighbor, military, rebels, other) of GBV; 3) locations where GBV occurred; and 4) barriers and challenges to reporting/seeking care for GBV. Specific attention was given to understanding how GBV occurred during conflict and subsequent displacement. Survivors and services providers were not asked to speak of their personal experiences but were asked to speak of GBV among IDPs, in general. Most survivors, however, did speak of their own personal, lifetime experiences. The focus group discussions with service providers included an additional area of focus related to service provision, including perceived and real barriers/challenges (e.g. time constraints, concern with asking sensitive questions, fear of offending clients, opinions on quality of services and lack of training) to implementing a GBV protocol in health care and other settings. Interviews and focus group discussions ranged from 50 to 90 minutes in duration.

Interviews were aided by locally-hired, trained interpreters with experience in GBV-related research to provide immediate interpretation during interviews and discussions. Written summaries were drafted at the end of each interview day and discussed amongst team members to debrief and identify gaps, new findings, or any issues that arose during research activities. These summaries also aided qualitative analysis.

### Analysis

Audio recordings were transcribed in the United States by a certified transcription company. All English transcripts were entered into Atlas.ti software (Cincom Systems, Berlin) for coding. Data analysis used pre-specified codes based on qualitative guides and research objectives and also followed a grounded theory approach, allowing for in-depth exploration of additional emerging themes in the participants’ narrative responses [29]. Each transcript was coded by a research team member (AW) trained in qualitative analysis and followed similar methods to those used for associated research [18]. Topical codes were applied to allow quotations to be sorted according to interview guide domains and open interpretive coding was utilized to identify and analyze any emerging themes observed within and between topical areas.

Findings present types and contexts of GBV during conflict and displacement in Colombia. Quotations were selected to highlight themes emerging from the analysis; some quotations provide information about one theme in particular, while others may provide information relevant to several themes. Quotations are labels with codes to indicate whether the quotation comes from a survivor of service provider. However, given the ongoing conflict, we have removed the names of the locations from individual reports as well as names of organizations to protect the anonymity of participants and service providers. Identification numbers are listed below quotations for the purpose of differentiation but are unlinked from individual participant origin. Names of armed groups were also excluded from quotations to further protect participants. Additional supporting quotes are provided in the appendix and referenced in the following text as: Additional file 1.

### Human subjects protection

Research and ethical approval was granted by the Colombian Ministry of Social Protection and the Johns Hopkins Medical Institute (NA_00049747). The research protocol and methods are consistent with Institutional Review Board guidance as well as the WHO guidance on researching sexual violence in emergency settings [30].

## Results

Survivors and service providers discussed GBV in the context of two distinct environments: the rural, conflict setting and the host area(s) to which the survivor and family were displaced. Few types of GBV were specific to only one setting. In the conflict setting, women were subject to forced recruitment and labor; early or forced marriage; ongoing threats and multiple displacements perpetrated by armed actors. IPV and intrafamilial violence were reported across both settings. In the displaced setting, IPV was the dominant form of reported violence, though other violence included abduction and sexual violence, often perpetrated by strangers encountered in town. Table 2 summarizes the types of violence, perpetrators, and locations in which violence occurs, within these settings. These experiences of violence were often linked to vulnerability during conflict and displacement, power dynamics and gender norms within relationships, and changing social and economic well-being. One provider summarized the fluctuating experiences and vulnerabilities associated with conflict and displacement in Colombia.

**Table 2:** Contexts of GBV during conflict and in displaced settings in Colombia

	**Conflict setting**	**Displaced setting**
**Violence types**	- Threats of violence, against family and well-being	- Threats of violence, against family and well-being
	- Forced recruitment*	- Opportunistic violence*
	- Physical violence	- Physical violence
	- Sexual violence	- Sexual violence
	- Forced abortion *	- Intimate partner violence
	- Intimate partner violence	- Reproductive control
	- Reproductive control	- Violence during pregnancy
	- Violence during pregnancy	- Intrafamilial violence
	- Intrafamilial violence	
**Perpetrators**	- Armed actors: guerrillas, paramilitaries, military*	- Strangers*
	- Intimate partners	- Owners of residence where staying*
	- Family members or in-laws	- Intimate partners
		- Family members or in-laws
**Locations**	- During recruitment/in guerilla camp	- On the street
	- In community during conflict	- In residence where staying/renting
	- Home	- Home

*Specific to respective conflict or displaced setting only.

*When the armed conflict arrives, she (woman in conflict setting) has to leave. She has to emigrate. But some have to stay and give up their sons to the armed conflict with the hope that this way she can maintain contact with them - even if they go far away she knows where they are. So that she doesn’t have to suffer coming to the city and go through hunger and needs. So, these women continue to live a process of psychological violence. This is a different kind of violence: not only is there vulnerability when they’re displaced but also when they choose to stay within the conflict. And the women that do displace themselves continue to be vulnerable as objects to violence from new relationships. They arrive here, another guy comes and another guy and another guy. Sometime this is due to economic necessities because they have no income. –*Provider FGD01

Below, we describe the contexts and types of GBV experienced by Colombian women in the conflict setting and in displacement. Results conclude with findings related to GBV service provision and barriers to care.

### Conflict setting

One of the most salient descriptions of violence in the conflict setting were reports of threats to life and safety, restricted movement, and threats to well-being of women and their families. Threats were a tactic to achieve social control of a community, recruit civilians to the armed group, and/or support the needs of the guerilla groups, paramilitaries, and/or military (Additional file 1, quote 01). Often, communities received serial threats by one armed group after another, each accusing the community of collaborating with other armed groups. Several participants described the general context of violence perpetrated against civilians by armed combatants.

*In [the first village] two groups were fighting and that was what caused the displacement. Both groups belong to [name redacted] Guerrillas. It was a fight between them. We were forced to leave because our houses were burned. So we left everything. The [second] village was taken by the guerillas, and then the paramilitaries came. The guerilla boss told us that if we stay after paramilitaries, they [guerrillas] will consider us, our lives, as targets. Then, the paramilitaries came to the whole region. So, we came to San Jose and to live in one room with five kids.* –Survivor 101

In some cases, threats followed participants after displacement.

*And eight days afterwards, they sent me that plastic bag, my little brother-in-law’s fingers. And that they had burned down a house and that they had taken all the cattle and all the cows and all the animals that we had. And they sent us papers saying that if they ever saw us again that they would kill us.* –Survivor 104

These reports of violence in the conflict setting predominantly arose from the interviews of participants whose displacement was relatively more recent compared to those whose experiences of displacement and/or conflict had occurred well in the past. For those whose displacement was relatively more distant, experiences of GBV in the urban setting were most strongly recalled. In these contexts, early or forced marriage was also related to guerilla groups who recruited young women in rural areas. Several women reported being displaced as a result of their attempts to protect their daughters from being recruited by guerillas.

*A few days went by and again I found another letter for my daughter. And it said that 11:00 P.M. they were going to expect her and “be careful; don’t leave us waiting. If you want more money, we’ll give you more money”. And my daughter, she had a friend the same age [12 yrs] and in the letter, it said the name of the friend and it said, invite that friend of yours, tell her “she can also come with us, bring her, and come to this certain place…” And so a lady [whose husband was in the guerilla group] told me, “you have to leave. They [guerillas] told me, you have to leave now; I already found out the information; if you don’t take your girl, they are going to take her and they’re going to take her by force”. –* Survivor 109

While threats of violence and forced recruitment and kidnapping were the most commonly reported events in the conflict setting, some extreme cases included forced labor, physical violence, and rape. One participant recounted her experience of being forced to labor for the guerillas, followed by attempted gang rape, and forced abortion.

“*My displacement, I lost a seven-month year old baby girl. It was forced abortion, military forces forced my abortion. So they forced me to make ‘picas’ - That means like little ropes within the jungle to be able to walk through those little trails. I was six months and a half pregnant… They took me to a camp where they already had some kidnapped people. They took me to an area where it was just like trees and grass. One of them tied me by hands and by my feet. They were all covered up with their uniform. She [female guerilla] was also holding my hands. She would hold me and the two other men raped me, two militia men raped me. I could see that she was crying… I tried to not let them because I was seven months pregnant, and since I didn’t let them rape me, they cut me open. The woman that was upset and she said she didn’t want to participate in this and decided that the commander hadn’t given that order, that that was not what was instructed to do. So she let me go. I was going to leave, and they shot her, they shot her in the leg. I tried to stand up as best as I could, but since they cut me deep, they cut all my genitals. –*Survivor 104

Beyond armed groups, intimate partner violence and intrafamilial violence when living in conflict areas was also widely reported (Additional file 1, quote 02). Several cases of IPV occurred during pregnancy. In many cases, the guerillas served as local ‘law enforcement,’ offering little recourse for female victims of IPV (Additional file 1, quote 03).

*He [husband] used to beat me. He pulled me by my hair. He used to shoot at me with a gun, so I would have to run away from the house. So, after the beatings I would just run away and just live outside the house, like in the middle of the trees, with my boy. He’d kick my stomach when I was pregnant. But I was a very religious woman and asked God to help.* –Survivor 107

### Displaced setting

Intrafamilial violence and IPV were dominant experiences in the displaced setting, often including threats, physical, and sexual violence. Physical violence included tactics that ranged from hitting, punching, kicking, throwing/pushing, pulling by the hair, to violence with weapons including machetes, guns, and knives. In many cases, IPV was attributed to alcohol use, infidelity or perceived infidelity, power differences, and gender norms (Additional file 1, quote 04).

*“So I met my husband. He used to work for the Colombian army. He was retired at that time. I moved with him. We have four children. And I was 16 years old at that time. He was a male chauvinist man. He was 40. He was like my father. He gave me orders. He beat me. When he wanted to go have some drinks with his friends I asked him not to go and he hit me. And he punched me on my face. And then he hit me also with a machete. Because he was an army member he had a gun so he felt more power. Even when he was sober he did that. He always wanted to be the boss.–*Survivor 112

Several women attributed IPV as an outcome of displacement or loss of financial stability. Women also reported increased IPV when their husbands faced unemployment when displaced to a new urban setting and women sought employment or education to support their families. Other women discussed facing a choice of displacement when their families were threatened by guerillas or children risked recruitment or abduction. Often husbands and partners chose to stay with their farms, but were later displaced and joined their families in town. Several of these women reported subsequent blame and violence by their husband for their decision to move. One survivor, who had who faced attempted rape and forced abortion by guerillas, recounted her husbands’ torture and subsequent violent behavior after being reunited following displacement:

“*He [husband] stayed for two days tied up with his hand hanging from his wrist. One night at 1 a.m., he [husband] managed to escape. The police helped him come to this area. And since then, my home was destroyed. He would say “what’s the use of working? What’s the use of saving money?” He started drinking, beating me. …Because I “let them rape me”, I was a “disgusting, degenerate bitch” and the “reason that the baby died” was because of me…*S*ometimes when he was asleep, I don’t know if he was dreaming about what happened, but he would grab me from the hair and throw me to the floor. I would tell him that “all we’ve been through and that we managed to continue our lives together. We’ve done all this together. Please don’t treat me this way”.*-- Survivor 104

Other forms of GBV were manifested in partners’ control of a woman’s reproductive decisions. This included forced sex, forced abortions, partner control over contraceptive use, and significant levels of physical violence during pregnancy. In several cases, forced sex resulted in unintended or unplanned pregnancy. One woman attributed her daughter’s Down syndrome to substantial levels of violence she experienced during pregnancy:

*After one month of being pregnant I had an abortion threat [risk of spontaneous abortion/miscarriage] and I was bleeding all the time but he was beating me all the time. I was nervous all the time, and I wasn't able to work at home because the doctors told me not to, and my daughter's father was beating me all the time. And now my daughter has Down syndrome. I was scared all the time, and I was bleeding after the seven months pregnancy. And she's the only one with Down syndrome. –*Survivor 108

Women, however, also described tactics to overcome their partners’ control of their reproductive decisions (Additional file 1, quote 05).

*Women don't let them treat us that way anymore. We don't let them decide for us anymore. If they throw away our pills, then we'll go get the shot, and if they don’t accept the shot, then we’ll have the Norplant, or whatever it’s called, under our skin. And there are women that say, “He doesn’t even know I have the-- they don’t know that I have the intrauterine--” -* Survivor 213

Several women noted patterns of serial marriages, in which they were married successively to another violent partner after the end of a prior violent relationship. Vulnerabilities to violence and repeat IPV was attributed to age differences between partners as well as financial needs, particularly for women who were forced to support themselves and their children in a new urban setting (Additional file 1, quote 06). Financial disparities are also associated with trafficking and engagement in sex work, as reported by a few survivors. One survivor reported being tricked to meet a stranger who offered her a job but was subsequently sold into prostitution. For other women, engagement in sex work was not through trafficking, but was the only feasible opportunity to improve economic well-being while simultaneously caring for their children during the daytime (Additional file 1, quote 07).

*Normally, the people that have been displaced are quiet people. First, because they don’t have enough vocabulary to express themselves. And because of the separation that they go into inside the cities-- the separation inside the cities do not permit them to get to the point that a normal person would be inside the city. So the woman is very submissive to men…It’s normal for a husband to verbally abuse the woman or he hits her and mistreats the children. It’s a form of control.* -- Provider FGD 01

Intergenerational patterns of violence also emerged from these interviews. Women reported witnessing or experiencing violence as children and experiencing new violence as adults. Likewise, survivors reported observing patterns of violence and victimization among their male and female children as well, fearing that their children would suffer the same experiences in their adult lives (Additional file 1, quote 08). Many took efforts to prevent their children from experiencing the same events in their lives.

*I allowed him to hit me; I knew it would be like something that was going to be happening again. And because my mother was beaten also, and I was beaten also by my mother, I know that I have to stop the situation. So I learned that from experience with my mother and my mother’s partner who also hit her.–*Survivor 102

Other forms of GBV were related to abduction and sexual violence, often perpetrated by strangers encountered in town. In these cases, the survivors were typically young women and often traveling alone in the evening. Perpetrators often used knives, drugs, and threats to force the woman into submission (Additional file 1, quote 09).

*I was about to go out to have dinner with some friends when I was walking around the [name redacted] Bridge and out of nowhere, a man came out with a knife and he says that he wanted to ask me some questions and my answer was that if you want to ask me something you can do it right away, in this moment in this place. And after that I started walking and this man was threatening me with the knife. And so he took me to an abandoned house. And in that abandoned house he raped me and after that he told me that I had to wait for some time until he gets on a motorcycle to leave. –*Survivor 115

### GBV service and reporting

In the conflict setting, there were often minimal or no health or protection services. As described above, guerillas often served as self-proclaimed authority figures in controlled areas. Most participants rarely sought or received medical care for injuries or related trauma; for serious trauma, some participants reported traveling long distances to receive care in a nearby city.

In each displacement site (Quibdo and San Jose de Guaviare) participants reported a range of services available to address the needs of GBV survivors. Health services included medical exams and gynecologic, forensics kits for sexual violence, HIV and STI testing, contraceptives, and post-exposure prophylaxis for HIV prevention, available at the local hospital and/or clinic. Counseling and psychosocial services were available, as were legal and protection services. Specific services are provided by various government institutions and some non-governmental organizations (NGOs). Displaced persons, who are registered as displaced, are entitled to receive some assistance for the first few months of displacement and can access services through specific institutions (Additional file 1, quote 10). While these services are available in urban and non-conflict settings, it is dependent on the survivor to actively report a case of GBV or seek these services.

[When asked if women who have had sexual violence are tested for HIV].

Provider 1: If they go to the doctor, yes, but if they stay at home they don’t know about it. They just find out something like they get pregnant and then after that that they get HIV.

Provider 2: Victims of sexual violence have the right to an HIV test. Have the right to a pregnancy test and they have the right to an abortion, but that happens if they go to a doctor. If they stay quiet and they stay at home that won't happen.

Provider 3: Besides the HIV test, the pregnancy test and the abortion they have the right to emergency contraception. –Provider FGD 06

Use of health, protection and psychosocial services was variable among participants. Utilization was often limited to seeking one of the available services, e.g. a participant may seek health services for rape, but may not follow referrals to law enforcement or psychosocial services. Poor initial experiences and perceptions of the survivors regarding the quality and safety of services, served as barriers to follow-up care or utilization of other referrals for some participants. These barriers were often related to their own embarrassment and shame, concerns related to confidentiality and privacy, perceived inaction by the institution/provider, institutional delays, and/or stigmatization in the facility (Additional file 1, quotes 11-13).

*I stayed quiet because they point us out here. Because you go to those types of places and everybody starts pointing out, “Oh, that woman was raped. Oh that woman has AIDS”. I was scared of rejection from my family or from my boyfriend so I didn’t say anything, I stayed quiet. Many years later I went to Doctors Without Borders with a psychologist. I told the psychologist and she helped me a lot.* –Survivor 213

*When I was about to have a baby, he [husband] would force himself on me and I was terribly sick… I felt too embarrassed to go to the doctor to tell them. I cried by myself, but I couldn’t tell anybody what was going on. I was terribly embarrassed.* –Survivor 109

One service provider indicated that women who experience sexual violence may be at fault for how they dress: *It depends on how the woman is dressed. So if the woman is dressed in a certain way, then they start yelling things at her or abusing them, because she was dressed that way and she was teasing men to do it.* –Provider interview 113

Barriers to care and reporting were also related to concerns of violent repercussions (including against health workers who provided care for survivors), if the perpetrator were to learn of a survivor’s disclosure of violence. Additionally, hospital staff received threats from some perpetrators. In some cases, survivors of IPV had to provide subpoenas to their husbands, increasing risk of retribution, while other survivors felt pressured by the authority figures not to formally report the violence (Additional file 1, quotes 14-16).

*After that, I went to the district attorney. I went there to make a report and the person in charge asked me not to make any report. The person that was asking me not to make a report had been contacted by the taxi driver, the man that raped me. And after that, after this man [in the district attorney’s office] asked me not to do that [report]. I believe that this taxi driver like convinced the judge and after that the judge told me it would be better if you don’t sign the report. He was moved by the things that the taxi driver said. I was offered 50,000 pesos so that I wouldn’t make the report … everything is led by money here.* –Survivor 116

*When I went to report the physical abuse, they didn’t pay attention to me so I don’t think they’ll pay attention now. I went twice to the Fiscalia [prosecutor’s office] because the aggression continued and-- the last time I went they sent the citation subpoena like three months after. When he found out about the citation he went to look for me and he beat me up completely. Then I had to go take away the statement, take it back or he would kill me…Then he started being involved with the armed groups and he told me he would stop bothering me if I didn’t proceed with suing him.* –Survivor 213

For survivors of IPV, the fear of breaking up family or losing financial support that is provided by the husband/partner often outweighed the fear of violence and perceived benefits of reporting or seeking justice for the violence (Additional file 1, quotes 17-18).

*…they never say, “My husband is beating me.” [Why is that?] Scared, they are afraid. And if you have kids and your husband leaves you and you don’t have a job, how are you going to support your children? Women with children won't get away from their husbands when they get abused because they are ones with financial support or the ones who pay the bills. –*Survivor 212

Despite the multiple individual and system barriers, participants disclosed or reported their experience to others with success in exiting violent relationships. Importantly, however, ending the relationship did not end the fear and trauma experienced by the violence.

*He [husband] did beat me up a lot. So I sued him and reported it to Bienestar Familiar, and the problems got worse, because he tried to kill my daughter and me. He shot me twice. He shot me twice with a rifle, and I sued him. I reported him to the Fiscalia, to the district attorney’s office, and now he has a restraining order. He can’t come near me, but anyway I’m still afraid.* –Survivor 208

When asked about potential facilitators to improve access to and uptake of services, survivors indicated that bringing the ‘human aspect’ to health services, demonstrating compassion, and ensuring privacy would improve survivors’ perceptions of health providers. Many indicated that they would willingly tell providers about their GBV experiences if the provider simply asked. Providers, on the other hand, indicated that improvements in resources and infrastructure would enable more effective and private provision of services.

*There are two needs they have in the hospital, for example. The first one is that they don’t have all the materials and the resources that they need, and the second one is that there’s no way to guarantee privacy for the victim, because when the victim goes to the doctor in the hospital maybe she will be facing other patients. So they don’t have an intimate room to have all the medical examination. –*Provider FGD 05

## Discussion

This study documents a wide range of GBV perpetrated against women in context of ongoing conflict and displacement, or multiple displacements. Armed combatants may perpetrate GBV that is related to conflict but GBV experiences by these survivors appeared to occur across conflict and displacement and is perpetrated by a range of actors. Perpetration of GBV may be augmented by new vulnerabilities attributable to displacement: economic and educational disparities, post-traumatic stress following experiences of violent events, loss of social support, changing gender roles, and loss of financial support either through loss of own employment or loss by the husband. For some women, these disparities affect decisions to stay in partnerships with violent men, become involved in new unhealthy relationships, and possibly engage in sex work for financial stability.

A dominating theme emerging from this research is the high levels of GBV, particularly IPV, which is generalized within the communities and appears to be exacerbated by the conflict and displacement. In fact, IPV among conflict-affected populations may be more common than conflict-related sexual violence (i.e. that which is perpetrated by armed combatants), while living in insecure economic situations and the resulting stress may further aggravate violence within the family structure [7,18,19]. This is supported by research from the general population of reproductive aged women in Colombia, including research from the 2010 Demographic Health Survey (DHS), which demonstrates substantial levels of violence against women, including non-consensual sex, physical violence by someone other than a current husband/partner, and lifetime IPV (physical or sexual violence) [31]. Despite high levels of violence and resulting physical or psychological health outcomes among Colombian women, health seeking behaviors for care following GBV was low [31]. Research from the Latin America region has also demonstrated increased associations of physical and sexual IPV and unintended pregnancy and abortion compared to pregnant women who had not experienced IPV [32]. Violence and displacement, often results in trauma, social disruption, experiences of job loss by partners, poverty, changing gender roles, and general frustration; these factors appear to exacerbate existing levels of IPV that exists in non-conflict settings for these displaced populations in Colombia [33].

This research highlights potential patterns of intergenerational effects and transmission of violence. Survivor participants reported witnessing or experiencing violence as children and experiencing new violence as adults. Other survivors reported observing patterns of violence and victimization among their male and female children as well, fearing that their children would suffer the same experiences in their adult lives. Exposure to violence during childhood is a known risk factor for future perpetration or victimization and witness of parental violence has demonstrated increased odds for experiencing violence within a woman’s lifetime in Latin America [34]. Exposure to armed conflict may further exacerbate these outcomes and other indicators of well-being among children and families. While the cross-sectional, qualitative design of this study limits such temporal inferences, other research among conflict-affected populations in Uganda has demonstrated the interfamilial patterns of violence among conflict-affected communities. In a two-generational study of families in conflict affected villages, the strongest predictors of self-reported aggressive parenting behaviors towards children were the guardians’ own experiences of abuse during childhood; female guardians’ victimization experiences in their intimate relationship; and PTSD and alcohol-related problems among male guardians [35]. In a similar study of conflict-affected populations in Uganda, a woman’s prior exposure to war-related traumatic events and alcohol-related problems among men were associated with higher levels of IPV against women [36]. Taken together, these findings demonstrate that action to care for survivors and prevent ongoing violence is vital to addressing and changing the trajectory of GBV among conflict-affected populations.

Recent media attention on events in Colombia contributes to the perception that the conflict may be resolving. In October 2012, the government commenced discussions with the leadership of the FARC, offering an opportunity for peace. No final agreement has yet been reached at the time of writing of this manuscript and the number of armed groups that exist within Colombia suggests that displacement may not end even if or when an agreement has been reached with the FARC [27,37,38]. In fact, 61 large group displacements were recorded between the months of January and July 2013, alone [26], while aerial bombardments, ground attacks on FARC operations, and bombings perpetrated by the FARC have continued into January of 2014 [39,40]. Our findings document threats and perpetration of violence against civilians caught in the geographic crosshairs of the armed conflict and is consistent with other human rights reports [41]. Trauma and mental health research of populations exposed to conflict in Colombia has demonstrated heighted odds of depression, somatization disorder, alcohol abuse, and anxiety-related psychopathology, compared those who did not experience direct violence [42,43]. This suggests that international attention and efforts by the government, NGOs, and humanitarian actors towards protecting and responding to the needs of communities in conflict settings and displacement should not wane at the promise of conflict resolution. The sustained conflicts combined with reports of generalized violence against women highlight the need to recognize the patterns of GBV across contexts and develop mechanisms to efficiently and effectively address GBV such that those who are most marginalized are also able to access quality and confidential care. Understanding the overall context of violence across conflict, displacement and integration into the host communities is critical for properly addressing GBV among IDPs in each phase of transition as well as to considering issues that may affect the host populations.

While the research objective was to specifically understand the context and types of GBV experienced by women in conflict and displacement to inform programmatic and policy response, we noted substantial agency and resilience among the women interviewed. Participants often took bold actions to move themselves, and their families, out of conflict or away from violent partners and prevent future victimization or perpetration of GBV among their children. This often placed women in unfamiliar settings where they faced economic disadvantages. Participants found ways to support their families, despite disadvantages and engagement in some less than favorable jobs. In cases of reproductive control by partners, women found ways to discretely access contraceptive methods or tubal ligation. Not only does this resiliency lend to individual or family-level survival and protection, but it can be and has been harnessed for response to GBV both in Colombia and among other conflict affected populations. Many of the Victims’ Laws for displaced people and laws preventing and responding to GBV have been advocated, monitored, and/or supported by community groups for survivors or displaced women in Colombia [44]. In humanitarian settings, individuals who have shared experiences can serve to provide support groups for survivors, provide peer education, and engage in community mobilization in others [45-47]. Ultimately, these women can play an important role in prevention and response to GBV.

This article should be reviewed in light of several limitations. First, we used purposive sampling to enroll internally displaced women who were survivors of GBV, residing in Quibdo or San Jose de Guaviare and surrounding regions, and receiving/had received psychosocial support and other care for experiences of GBV. While this is a commonly used sampling method for qualitative research and is the most ethical way to conduct such research and minimize both risk of secondary trauma and breaches of participant security, our understanding of experiences of those who never reported GBV/sought services or those who returned to living in active conflict areas is limited. Results are potentially subject to recall bias: participants who had been displaced for several years or experienced chronic or more traumatizing violence during displacement were less likely to discuss distant experiences that occurred during conflict. This may partially explain heterogeneous findings from Quibdo and San Jose de Guaviare, in which there were differences in recall on violence experienced in displaced settings compared to recall of conflict-related violence that may have occurred more distally in time for some. Nonetheless, our findings related to the conflict setting are supported by research and NGO reports, including for example, child recruitment, threats, physical violence, torture and dismemberment [48,49]. This study ascertained qualitative, lifetime experiences of GBV, so cannot provide temporal associations of which forms of GBV are associated with conflict or were more common in the past. Similarly, these findings should not be viewed as estimates of prevalence of GBV in the regions. We were also unable to interview young women between the ages of 15-18 years, despite evidence of need for inclusion of children in violence research. This was due to regulations that require parental consent to participate in the research; thus, we chose to enroll women aged 18 years and above instead, acknowledging that parents or family members may be perpetrators of violence. Research on GBV related to conflict and displacement is certainly needed for adolescents given their increased vulnerability. Interviews among survivors of this study, however, still provided important retrospective descriptions of GBV that occurred during childhood and adolescence and observation by survivors who also witnessed GBV targeted towards their children provide perspective of GBV experiences, the potential impact of exposure to violence on children, and future perpetration and/or victimization. Nonetheless, many participants reported substantial accounts of experiences of violence and delays to reporting, allowing great insight into shared experiences of violence, barriers to seeking services and reporting experiences to authorities. Finally, as with research that is not population-based, the generalizability of our sample to broader conflict-affected populations, both within and beyond the country, is unclear. However this study provides important insight into the causes and context of GBV in this setting. Such causes and contexts may also inform and provide insight into the prevalence of and response to GBV among other conflict-affected populations.

## Conclusion

Though the context of conflict, displacement, and GBV in Colombia has received less attention than other conflicts, there is a great need for continued political and humanitarian response in the country, particularly in matters related to GBV. The case of Colombia also serves as an important lesson for other conflict affected populations, recognizing the globally high levels of displacement induced by conflict, worse health outcomes of IDPs compared to other conflict-affected populations, and the recognition that future programs will need to provide services to displaced communities as they are integrated or ‘lost’ within host communities [50]. The Colombian government’s successes in legal and policy reform to address GBV in conflict and protect the rights of GBV survivors and women in conflict [51,52], along with new transitional solutions implemented by humanitarian organizations that seek to create sustainable solutions to address GBV and other needs among both host and IDP communities in Colombia [53], may serve as lessons for other countries and may be adaptable to other conflict-affected settings.

With respect to response to GBV, our findings highlight the need for early identification of GBV experiences, with emphasis on confidential approaches and active engagement of survivors to available, quality services. Building on these findings, our future research seeks to develop a screening tool for use by humanitarian agencies, government and NGO service providers, to identify women from the IDP population who have experienced GBV and offer referrals for care. Effective use of such a screening tool, however, requires availability of a minimum package of services to address physical trauma, adverse reproductive and mental health outcomes, and protection needs. Given the findings of concerns related to services in Quibdo, this setting was not a candidate for immediate future study of screening, and great effort is given to carefully identifying locations where confidential services are available. Settings where subsequent research on screening implementation was planned included San Jose del Guaviare and Mocoa. Much like IPV screening that is implemented in US healthcare settings [54], routine and confidential use of this tool may enable early identification of women in immediate need of health care and protection and may identify women who have previously failed to access services. In addition to early identification, over time the tool may reduce perceived stigma and concerns about health care personnel of GBV among survivors, may reduce community tolerance of IPV, and may ultimately facilitate the achievement of the objectives set forth by new laws to protect women from violence.

## Competing interests

The authors declare that they have no competing interests.

## Authors’ contributions

AV, LR, NG, AW, KP, SS conceived of the study and designed the protocol. DC participated in the management of field level data collection. SL and TK facilitated collaborations with Colombian Ministries, local organizations, and UNHCR field offices and provided input into Colombia specific field protocols and site selection. AW and GJ (acknowledged) led in-depth interviews; KP led focus group discussions. AW conducted qualitative coding, and analysis, interpreted of the results, and drafted the manuscript. All authors have read, provided input, and approved the final manuscript.

## Authors’ information

Qualitative research presented here is part of a multi-phase study conducted by the research team (NG, KP, LR, AW, SS, AV) to develop and validate an evidence-based screening tool to identify and refer female and male survivors of sexual and gender-based violence in humanitarian settings. The tool is being developed among refugees and displaced populations in Ethiopia, Colombia, and Uganda with the ultimate aim for international use by UNHCR and other providers to confidentially identify and meet clinical, reproductive health, and mental health care needs and provide protection for survivors.

## Supplementary Material

Additional file 1 Web Appendix. Click here for file
