# Supplementary material for: Gender-based violence in conflict and displacement: qualitative findings from displaced women in Colombia
Source: Confl Health. 2014 Jul 11;8:10. doi: 10.1186/1752-1505-8-10 (PMC4115473; doi:10.1186/1752-1505-8-10)
Supplement: Additional file 1 — Web Appendix. [file 1752-1505-8-10-S1.pdf]

Gender-based violence in conflict and displacement: Qualitative findings from displaced women in Colombia

Additional File

Authors:
AL Wirtz[1,2], K Pham[1,3], N Glass[4], S Loockhart[5], T Kidane[5], D Cuspoca[6], LS Rubenstein[2], S Singh[2,], A Vu[1,3]

Affiliations:
1 Department of Emergency Medicine, Johns Hopkins Medical Institute, Baltimore, USA
2 Center for Public Health and Human Rights, Department of Epidemiology, Johns Hopkins School of Public Health, Baltimore, USA
3 Department of International Health, Johns Hopkins School of Public Health, Baltimore, USA
4 Johns Hopkins School of Nursing, Baltimore USA
5 United Nations High Commissioner for Refugees, Bogota, Colombia
6 Departamento de Antropología de la Universidad de los Andes, Bogota, Colombia
7 Department of General Internal Medicine, Johns Hopkins Medical Institutes, Baltimore, USA;

Email Addresses:
Co-author email addresses: A Wirtz (awirtz@jhsph.edu); K Pham (kpham4@jhmi.edu); N Glass (nglass1@jhu.edu); S Loockhart (LOOCHKAR@unhcr.org); T Kidane (kidanet@unhcr.org); D Cuspoca (djcuspoca@gmail.com), L Rubenstein (lrubenst@jhsph.edu); S Singh (ssingh31@jhu.edu); A Vu (avu3@jhmi.edu)

Table 1 Additional relevant quotations from internally displaced female participants in Quibdo and San Jose de Guaviare, Colombia, stratified by topic discussed in the results of manuscript. All quotations are marked with a sequential number (left column) to locate the associated text in the manuscript.

**Conflict Setting**	
No.	Quotation
01	*Participant 5: In other cases, as well the woman is forced to join the illegal groups, where at that moment she had suffered violence, sexual violence and psychological violence.*<br>*Participant 6: Emotional violence. We have within the conflict they have sexual, physical.*<br>*Participant 2: Recruiting is the worst thing when they force them to go into the illegal forces.*<br>*Participant 5: They force the children and the husband to go into the conflict. And if they don't do it, they kill them.*<br>*Participant 7: And the young girls are also forced to join the illegal services, the illegal forces. And if they refuse to go, they are killed. Or if they are able to escape then they become displaced.* -- Provider FGD 01
02	*Once I found out he [husband] was dating a woman. His brother's wife told me and he thought it was a man who told me…Out there he hurt me with a gun and he wanted me to tell him who was the one who told me about the other woman. I wouldn't tell him that it was his brother's wife, the one who told me. I went back to the village and he was following me and he started running after me and so he grabbed me and started yelling at me. He has hit me with his gun and I told him, because I was afraid. I told him it was not a man who told me. It was your brother's wife, so he had let me go. So he kicked out my brother's wife, who was living with us at that time with them. At the time there was not any authority or not law enforcement or-- there was not anywhere or anybody who you can talk to or report this situation. The only authority over there was the guerillas. The guerillas hear about my problem with my husband and they ask me what happened. They told me that he was right, that there were not many women who had ten kids and could raise them, alone, without his economic support.*<br>–Survivor 101
03	*For example, the case I'm working on right now is a girl that, since she was eight years old, her brother-in-law has been abusing her…he would force her to watch how he raped other women. And if she turned her face or looked another place, he would slap her until she continued staring to where the act was happening…When he started abusing her, she was 10 years old, physically abusing her. When she was 11, she got pregnant from him. He got her pregnant. Every time he would get her, it was by force, and now she has three children, all belonging to him. She lived in this rural area. The guerilla are in control there. So the guerilla came to him and told him he had to choose. He couldn't be sleeping around with all these girls, that he had to pick only one of them- of two sisters, and the other one was their cousin. So he chose the other sister. So later, she was displaced from there to Quibdo when she was 15 years old, and she already had the three children.* – Provider interview 113
**Displaced setting**	
04	*Well, he would come home and he would insult me and beat me and tell me I had to have sex with him, and even though I didn't want to, after a while of him beating me, I was so tired of him beating me that I would say, "Okay, let's have sex, as long as you stop beating me." …He would kick me out of the house and wouldn't let me go back in, but the girl [daughter] was inside the house with him, and he probably was drunk, and he wouldn't let me get the girl out of the house. So he said he would only let me come into the house to get the girl if I had sex with him, and he would force me to have sex with him.* –Survivor 110

**Displaced setting**	
No.	Quotation
05	*He [husband] never let me use contraception. I always wanted to have just two kids but because he didn't let me use any contraception - that was why I had four. And he didn't let me get sterilized... When I had my last kid, who was a girl, I had a health problem. They referred me to a hospital in Villavicencio and then I took advantage of the situation and asked the doctor to sterilize me.* –Survivor 112
06	*With my first husband, I had a very miserable life. He would beat me up very often and I moved in with him when I was very little. I was 16 years old. He would beat me, he would humiliate me. He would make me run from house in the middle of the night because he was going to beat me. He was beating me because I didn't want to be with him and he would come home drunk. I believe that a man that is drunk and forces a woman-- himself on a woman, I believe that that's called rape. I prefer him beating me or I would run away from the house than to be raped. Doesn't matter if I was carrying the baby, the little baby girl. He would throw me to the floor anyway. I lived for five years with him, until I couldn't handle anymore and I left the house. Then I got together with another husband-- same thing all over again.* —Survivor 110
07	*My friend took me to the bar and then she told me what was the job about and told me that it if I wanted to work as a prostitute I could do it or I could find another job like a domestic worker in a house. But, she told me that it was better to have this kind of job because it was at night time and at that time her kids were sleeping so it was okay to be out of the home... I always tried to get out of that kind of world because she always thought it was terrible… when I see some other sex workers I feels their pain inside and feel sorry for them…And, the biggest sacrifice was to hide that job from my kids.* –Survivor 103
08	*I always ask him not to do it [abuse the children], but once he hit my daughter so bad that I had to take the girl to the hospital. I said I always defended my children, but that time when he was hitting my daughter, he hit me too. He punched her [daughter]. He kicked her. He pulled her by her hair. She was nine years old… Because of all that mistreating is why my children are so scared of doing things. My two sons got the worst violence of all the children. The girl, the one that they got into the hospital, is a grown up woman, she failed in her marriage, and she told her husband that she didn't want to go through the same story that her mother went through. So she asked him not to yell at her and not to be mistreated by him…But it happens that when they didn't have enough money, so he started getting angry and arguing with her, because she wasn't making money. Now, she's living by herself with her kids and she's working. The boy who was kicked out by his father from Villavicencio, he's not here. He's just drinking. He has a bad temper and that he is a lot similar to his father.* – Survivor 112
09	*All I can remember was that somebody tried to stop the taxi and the next thing that I remember was that moment that I woke up and I was locked in the room and I was already abused. [Interviewer: So when you woke up, was the person still there?] Yeah, the people - there were two men there. When I woke up, I noticed that I was completely wet, and I started crying, so somebody heard and called the police.* – Survivor 116

	**GBV Service and Reporting:**
No.	Quotation

	**GBV Service and Reporting:**
No.	Quotation
10	*Participant 4: If she [a survivor] has been displaced, she takes the information and forwards them to the victim's section of the national government. Once she arrives at that point they will decide there whether she needs psychological help or healthcare or whatever. If she's not a displaced victim then ICBF takes care or the local family commission, which is dependent from the police really. That's what they do according to what we need.*<br>*Participant 5: The institution handles emergency services having to do with the initial emergency treatment, involving the specialist and interdisciplinary groups. Therapy. Orientation. And the case is forwarded to different institutions so that those people receive the appropriate help.*<br>*Participant 6: They implement the national laws according to people that have been violated and especially the health component. They supervise that the persons that have been in this situation receive the proper healthcare. If they [the survivors] are affiliated to a health services provided through EPS here these private organizations have to comply with the mandatory national plan for these people. If they're not affiliated to this kind of group, the health secretary using government resources-- they either have to be affiliated to these companies that offer the health services. The person, each individual has to be affiliated to these groups and if they're not then the government covers it. But, they insist as much as possible that these people become affiliated.*<br>–Provider FGD 01
11	*[After GBV] I received Nurses attention. The nurses' care was really fine to me, but I didn't feel comfortable at all with the female psychologist. Because the female psychologist mentioned that I had a relationship with the youngest man from the two [perpetrators], so I felt uncomfortable and that was not right because I didn't even know the man. The psychologist was blaming me for what happened.* –Survivor 116
12	*Almost the whole neighborhood found out about it [participant's experience of rape]. And now I've moved out and I'm living with my father. That's why when I go back there I'm always looking down, looking at the floor. And my neighbors there [back where it happened] don't come to me to support me but to ask me how was the rape, what had happened, things like that. So they try to revive the situation again.* –Survivor 119
13	*They [in the institutions] don't care what we're telling them.. You're saying, "Hey, I need an appointment, urgent, with my daughter." Then they get a cell phone and they say, "Yeah. Yeah. Wait a second. Hold on. Hold on." And they don't pay attention to us… Sometimes we go to ask for services, and so we explain what's happening and they say, "Yeah. Okay. Well right down your name. Come two weeks from now and I'll give you an answer." And when we arrive, the same person is not there. Somebody new is there and I have to start all over the process again.*<br>–Survivor 104
14	*Sometimes they [survivors of IPV] do report the situation. The thing is that they are not hearing back from the institution where they report it, and they are getting beaten again by their husbands. And what happens is that when they call their husbands for an appointment from that institution where the woman reported the situation, they give the paperwork to the woman so the woman can give it to her husband. So women feel very scared to report a situation, or sometimes they report it and then they just take it back. –Survivor 121*
15	*Interviewer: Have you ever sought any services when you were experiencing this? Participant: No because my mother-in-law threatened me that if I sued him she would kill me.*<br>–Survivor 202

	**GBV Service and Reporting:**
No.	Quotation
16	*I once went to the health center. Once he hit me so bad, my head, that I had to go there. I told the nurse. So, the nurse was pretty mad, saying, "Why is he treating you this way," and all this. And then my husband came into the office and the nurse told him, "Why are you mistreating your wife? Why are you beating her?" And my husband told the nurse, "Well, if you want to get hit, then keep talking to me." The nurse stayed quiet; she didn't say anything else.* –Survivor 107
17	*I've been living with a man for the last 10 years, but for the last year, year and a half, he's been hitting me and insulting me. I'm the worst person that exists in the world for him…. I went to the district attorney's office and I sued him. I can't take it anymore. I have three kids. They are six, four and two years old. He threatened me that, if I leave him, he'll take away the kids. That's why I spent this last year super sad and bitter. I don't know if I can call him my husband or not, but I'm living with him right now.* –Survivor 105
18	*Because the majority of this population they are completely illiterate…When they arrive to the city, even though we try to help them with subsidies since they have no education, it's very difficult for them to survive. They have no entrance work only the low, very low category jobs such as selling candy, selling food, selling prostitution. Or work exploitation such as coming into work inside homes as maids at a very low, low salary. And their sons since they have the same situation where they have no education then poverty starts to be created.* –Provider FGD01
